# Left behind and reimagined: identity reconstruction and the social burden of children’s migration on older adults

**DOI:** 10.3389/fsoc.2026.1896525

**Published:** 2026-10-30

**Authors:** Judith I. Ani, Kezia Batisai

**Affiliations:** Department of Sociology, Faculty of Humanities, University of Johannesburg, Johannesburg, South Africa

**Keywords:** identity reconstruction, international migration, left behind, older adults, transnationalism

## Abstract

As international migration reshapes family structures across sub-Saharan Africa, older adults left behind bear disproportionate social, emotional, and caregiving burdens that are unevenly distributed by gender and remain underexplored in migration scholarship. Drawing on qualitative in-depth interviews with 20 older adults in Edo State, Nigeria, and on the transnational social field perspective, this study examines how the international migration of children disrupts established care networks, triggers identity renegotiation, and reshapes the social construction of aging. Three interrelated themes emerged: migration as a social determinant reshaping care, well-being, and identity; migration as a socially constructed experience; and migration as a gendered phenomenon reshaping roles, identities, and vulnerabilities. Findings suggest that migration simultaneously disrupts proximate care and generates emotional distress, while remittances paradoxically elevate older adults’ community standing, enabling active identity reconstruction beyond passive loss. Older adults constructed migration as an economic necessity, navigating a duality of pride and loss, cultural negotiation, and spiritual meaning-making, with religious and community institutions emerging as critical surrogate support systems. Identity reconstruction was real but unevenly available: participants with access to remittances, transnational contact, and supportive institutions reimagined themselves as transnational parents and community benefactors, while those without such resources, particularly widowed women living with illness, carried the burden of migration without its compensations. We conceptualize these experiences as left-behind transnational parenthood: parenthood, care, and social standing reorganized from the origin side of the transnational field.

## Introduction

Migration prevalence across the globe is increasing, driven by a complex interplay of variables that extend beyond economic considerations alone (United Nations DESA, 2020). Neo-classical economic theorists posited that wage differentials between countries are the primary driver of migration (Todaro, 1969). This assertion has since been contested, given that both the most economically disadvantaged and the wealthiest countries record substantial shares of transnational populations per capita (United Nations DESA, 2020), albeit disproportionately so in developing countries. This implies that migration is shaped by factors beyond living standards and wage discrepancies, encompassing individual aspirations, preferences, and structural opportunities (de Haas, 2011; Kureková, 2010).

Nigeria is the largest recipient of remittances in sub-Saharan Africa. In 2023, it received an estimated US$19.5 billion, around 35 per cent of all remittance inflows to the region (World Bank, 2024). In origin communities, remittances pay for food, schooling, housing, and health care, and in many households, they have become an expected part of family income. Their effects reach beyond the household. In Benin City, having children abroad is widely regarded as a marker of family status (Akhigbe and Effevottu, 2023), and migration changes who in a community is available to provide care. For older parents, then, a child’s migration can bring both material gain and the loss of everyday support.

Globally, the number of individuals residing outside their country of origin or legal nationality surged to 281 million in 2020, up from 173 million in 2000 and 221 million in 2010. This growth in international migration outpaced the global population growth rate, with the proportion of international migrants relative to the total global population rising from 2.8 per cent in 2000 to 3.2 per cent in 2010, and further to 3.6 per cent by 2020 (United Nations DESA, 2020). Sub-Saharan Africa has long been characterized by significant levels of human mobility, a phenomenon predating the establishment of colonial-era borders. By mid-2020, approximately 7.6 million people had migrated within this sub-region (United Nations DESA, 2020), though this figure likely underestimates the true scale of movement given the inadequacy of existing data in capturing the extent of temporary and seasonal migration prevalent in the region. Notably, an increasing number of individuals are relocating to destinations within and beyond Africa, driven largely by the pursuit of employment and improved economic and social prospects. Simultaneously, the rising population of older adults across sub-Saharan Africa and other developing nations places mounting pressure on public and social institutions responsible for elder care - a challenge further compounded, in many contexts, by the absence of adequate institutional frameworks to meet the needs of aging populations (Ani, 2014; Batisai, 2017; Wong and Higgins, 2007).

The consequences of adult children’s migration for parents who remain have been studied most extensively in Latin America and Asia. In Mexico, the migration of adult children to the United States has been associated with poorer physical and mental health among older parents (Antman, 2010). Qualitative work there has documented the sadness, longing, guilt, and worry felt by parents of migrants, and found that mothers and fathers perceived and expressed these emotions differently (Fuller, 2017). Studies from Thailand, Nepal, Moldova, and Albania report similar losses, while also showing that remittances and continued contact can offset some of them (Knodel and Saengtienchai, 2007; Vullnetari and King, 2008; Adhikari et al., 2011; Böhme et al., 2015). A review of this literature found that several studies reported adverse effects on parents’ mental health (Thapa et al., 2018).

The phenomenon of international migration among children from sub-Saharan Africa is gaining momentum, with significant implications for the care, well-being, and identity formation of the older adults left behind. Existing scholarship shows that the departure of children to foreign countries can generate a range of adverse outcomes for older adults who remain, including emotional distress, loneliness, social isolation, mental health challenges, physical ill-health, and loss of instrumental and economic support (Adhikari et al., 2011; Ebimgbo and Okoye, 2024; Abas et al., 2009). Evidence from a Mexico-United States migration study established a causal relationship between adult children migrating to the United States and deteriorating health outcomes among older adults remaining in their origin communities (Antman, 2010). Similarly, research conducted in Bangladesh documented heightened vulnerability among older adults left behind by migrant children, arising specifically from the deprivation of personal support and caregiving (Kuhn, 2005).

Evidence from sub-Saharan Africa is much thinner. In Nigeria, recent studies in the south-east have documented the challenges faced by older adults with children abroad and the ways they rationalize their children’s departure (Ebimgbo et al., 2022; Ebimgbo and Okoye, 2024). Less is known about how being left behind changes older parents’ sense of who they are, or about how gender shapes these experiences. Identity in the migration literature has been studied mainly among migrants themselves (Jaspal, 2015). This study addresses these gaps in Edo State, one of Nigeria’s major origins of international emigration.

Trends in international migration among adult children have contributed to a decline in the availability of family-based care for aging parents—a particularly critical issue across Africa, where children constitute the primary source of support for older adults (Ani and Isiugo-Abanihe, 2023; Aboderin, 2004). This situation is made more acute by the growing number of women migrating, given that women bear the primary caregiving responsibility within prevailing gender structures in sub-Saharan Africa. Historically, women migrated largely as dependents of spouses or male family members; however, an increasing proportion now migrate independently for educational or employment purposes, with many assuming the role of primary breadwinner for their households. This shift is reflected in aggregate statistics, with women and girls accounting for nearly half (48 per cent) of all international migrants globally in 2020 (United Nations DESA, 2020). Traditionally, men from the region migrated alone in pursuit of employment while women remained in their countries of origin (Oucho, 2006). This pattern, however, is undergoing marked transformation, most visibly in the rising trend of women from sub-Saharan Africa migrating independently for work or study (Adepoju, 2000).

The rising trend of young adult migration has prompted critical questions about whether the absence of children from households and communities influences the well-being and identity formation—including health-seeking behavior—of older adults left behind in sub-Saharan Africa. Existing literature on migration outcomes has predominantly centered on the effects experienced by receiving regions and migrants themselves, as well as on the drivers of migration, including the 2020 Africa Polling Institute survey on Nigerian emigration to Canada (Africa Polling Institute, 2020). Comparatively little attention has been directed toward the consequences of migration for origin communities and the older family members who remain. Similarly, scholarly inquiry into identity formation and migration has largely focused on migrants at their destination (Jaspal, 2015; Sulyman, 2014), with a notable absence of studies examining identity reconstruction among left-behind older adults, particularly within sub-Saharan Africa. The health and social impacts of this phenomenon in the sub-Saharan African context, therefore, remain insufficiently understood. This study, therefore, examines how the international migration of children shapes the formation of new identities, care, and well-being among left-behind older adults in sub-Saharan Africa.

Some concepts organize this inquiry. The first, *social burden*, refers to the emotional, caregiving, economic, and decision-making costs that adult children’s migration imposes on the parents who remain. These costs are unevenly distributed and shaped by gender. The second, *reimagining*, captures the observation that older adults are not only acted upon by migration but actively rework who they are in response to it. Drawing on identity process theory (Breakwell, 1986; Jaspal, 2015), we understand identity as something continually revised when established sources of self-esteem, continuity, distinctiveness, and self-efficacy are threatened or newly made available. A child’s departure can threaten these sources by removing the everyday presence through which parenthood, provision, and companionship were enacted. Remittances, transnational contact, and community recognition can open new ones. The pairing of “left behind” with “reimagined” signals this dual movement. Older adults are left behind physically, yet many reconstruct their identities in its wake. Whether they are able to do so, and at what cost, is the question this study pursues.

*Care* is understood not only as hands-on assistance delivered in person, but as a set of practical, financial, emotional, and personal practices that can circulate across borders within transnational families (Baldassar et al., 2007; Baldassar and Merla, 2014). This framing lets remittances, phone contact, and visits be read as forms of care alongside co-resident caregiving. It also recognizes that some forms of care, such as personal care during illness, depend on physical presence. *Well-being* is understood as multidimensional, spanning physical, emotional, and social domains, and as the balance between a person’s available resources and the challenges they face (Dodge et al., 2012). On this view, migration can affect well-being in two directions at once: it removes some resources, such as companionship and daily help, while adding others, such as money for medical care.

The study’s aims connect to two policy commitments. The African Union’s Protocol on the Rights of Older Persons (African Union, 2016) calls on states to support families caring for older people. When the family members expected to provide that care have migrated, it is unclear what such support should entail. Evidence on how older parents experience their children’s absence is needed before that question can be answered. The 2030 Agenda’s pledge to leave no one behind (United Nations, 2015) similarly directs attention to groups whose needs are overlooked. Older parents of migrants are largely invisible in both migration data, which record those who leave, and aging data, which rarely record whether an older person’s children live abroad. This study contributes to these commitments by documenting the experiences of this group.

Specifically, this study explores how international migration among children reshapes the care, well-being, and identity of older adults in Edo State, Nigeria, and examines how left-behind older adults socially interpret their children’s migration. In doing so, it documents the social, emotional, and health-related challenges confronting this population and contributes to migration and aging scholarship from an African origin context.

### Research questions

The study was guided by three research questions:

1. In what ways does the international migration of adult children shape the care, well-being, and identities of older adults left behind in Edo State, Nigeria?
2. How do left-behind older adults interpret and make sense of their children’s international migration?
3. In what ways does gender shape older adults’ experiences of their children’s international migration?

The first question addresses the social burden of migration and its consequences for identity: how care arrangements, well-being, and older adults’ sense of themselves change when children live abroad. The second addresses meaning-making, including older adults’ place in decisions to migrate and the meanings they attach to their children’s departure. The third examines how gendered expectations in combination with widowhood, income, and health, shape these experiences. Together, the questions treat older parents as participants in transnational family life rather than as its residue, the perspective developed in the theoretical framework below.

### Theoretical framework

This study is anchored in transnationalism, specifically the transnational social field perspective on migration (Glick Schiller et al., 1992; Levitt and Schiller, 2004). Early migration theories treated migration as a one-directional move in which migrants left their societies of origin behind. Transnational scholarship instead showed that migrants sustain economic, social, and emotional ties across borders, linking origin and destination within a single social field (Glick Schiller et al., 1992). Crucially, this field includes people who never move. Those who stay are embedded in transnational relationships through the remittances, communication, obligations, and expectations that cross borders (Levitt and Schiller, 2004). From this perspective, older parents in origin communities are not residual figures left outside migration but participants in it, whose lives are reorganized by flows they do not control.

Transnational migration is also deeply gendered. Mahler (1999) argued that transnational research had neglected gender, and that gender shapes who migrates, who stays, how ties across borders are maintained, and how relations within origin households are transformed. Subsequent work extended this into a framework for analyzing how gender operates across household, community, national, and transnational scales (Pessar and Mahler, 2003). Feminist scholars have further cautioned against treating gender as a single binary category, arguing that it must be analyzed in its intersection with class, age, marital status, and place, and across national contexts (Kaplan and Grewal, 1994; Mahalingam et al., 2009). Mahalingam et al. (2009) show that motherhood, in particular, is not a universal experience but is shaped by intersecting social positions and idealized cultural expectations that travel across borders.

Within this literature, research on transnational families has documented how parenting and care are sustained across distance. Studies of transnational motherhood have shown how migrant mothers continue to parent children left in their countries of origin (Hondagneu-Sotelo and Avila, 1997), and work on transnational care has shown how practical, financial, and emotional support circulate among family members across different countries (Baldassar and Merla, 2014). Much of this scholarship, however, centers on migrants as the agents of transnational family life, and particularly on migrant parents caring downward for children they have left behind. The position of older parents who remain in origin communities while their adult children migrate, a position in which care and support flow upward across borders, has received comparatively little theoretical attention (Toyota et al., 2007). This gap is especially marked in sub-Saharan Africa.

This study addresses that gap by examining left-behind transnational parenthood, that is, the experience of older parents whose parenthood, care, and social standing are reorganized from the origin side of the transnational field. Two concepts structure the analysis. The first, social burden, refers to the emotional, caregiving, economic, and decisional costs that participation in a transnational family places on those who stay. Because gender and other social positions shape where people stand within the household and community, these costs are distributed unequally (Mahler, 1999; Pessar and Mahler, 2003). The second, reimagining, refers to the ways older parents renegotiate their identities and standing through resources circulating within the transnational field, such as remittances, contact, and the social recognition attached to having children abroad (Levitt, 1998). Care is understood throughout as a set of practices that circulate across borders (Baldassar and Merla, 2014). Well-being is understood as multidimensional, reflecting the balance between a person’s available resources and the challenges they face (Dodge et al., 2012). Taken together, these concepts allow the study to ask not only what migration costs older parents, but how, and for whom, it opens new ways of being a parent, a woman or man, and a member of the community.

## Methodology

### Study design

This study adopted a qualitative design to examine how the international migration of adult children shapes the care experiences and identities of older parents who remain in Edo State, Nigeria. Research on parents left behind has been predominantly quantitative, measuring outcomes such as physical and mental health (Antman, 2010; Böhme et al., 2015; Thapa et al., 2018). Such studies establish that migration affects parents, but they are less able to show how parents interpret their children’s departure, how they reorganize care, and how they come to see themselves differently. These questions concern meaning and self-understanding, and they are best addressed through participants’ own accounts. A qualitative design was therefore chosen, with interpretative phenomenological analysis (IPA) (Smith et al., 2022) guiding the analysis.

### Study area

The study was conducted in Nigeria, with Edo State purposively selected as the study site because of its well-documented history and contemporary significance as a site of international migration, particularly migration to Europe (Tuki, 2025; Beber and Scacco, 2022; Vermeuelen, 2019). Benin City, the state capital, has been identified as a migration hotspot. It is widely recognized as one of Nigeria’s main hubs of emigration and remittance receipt, and having children abroad is widely associated with family status (Akhigbe and Effevottu, 2023).

Evidence from a survey conducted in the city found that 23% of households reported that someone had left in the previous year to “follow land,” a locally used expression for irregular migration along the Mediterranean route; this accounted for approximately two-thirds of those who had left intending to reach Europe. Approximately one-third of randomly sampled respondents also reported having a family member abroad who had traveled by such routes, most commonly to Italy (Beber and Scacco, 2022). These findings illustrate the substantial scale and social salience of international migration in the setting.

Geographically, Edo State lies in Nigeria’s South–South geopolitical zone. Its main ethnic groups include the Bini, Esan, Afemai, and Owan peoples, and Christianity is widely practiced alongside indigenous religious traditions. Family life is organized around patrilineal extended kin networks. Obligations of support reach beyond the parent–child relationship to siblings, in-laws, grandchildren, and lineage members, and sons, particularly the eldest son, carry specific ritual and inheritance responsibilities. Care for older people has therefore customarily been spread across this wider network rather than resting on any single child. Women have featured prominently in migration from the state to Europe. In this setting, the migration of children is both common and socially meaningful, and its consequences for older parents are shaped by the extended family system within which care is organized.

Therefore, Edo State is a particularly relevant context for examining the consequences of adult children’s international migration for older parents who remain behind. The state’s established transnational migration networks, combined with economic precarity and family structures in which intergenerational support is embedded within extended kinship networks, provide an important context for examining how migration-related family separation may affect older adults. Importantly, the present study does not treat all youth out-migration from Edo State as international migration. Rather, participants were purposively selected specifically because they had at least one adult child who had migrated internationally.

### Inclusion criteria

Participants were selected using purposive sampling. Eligible participants were (i) aged 60 years or older, (ii) resident in Edo State, and (iii) parents of at least one adult child who had migrated internationally.

### Sampling

Participants were selected through purposive sampling to recruit older adults with direct experience of children’s international migration. Recruitment was facilitated through community networks and peer referrals to achieve diversity across key socio-demographic variables, including gender, socioeconomic status, and geographic location within the state. Deliberate efforts were made to include participants from both rural and urban communities. This sampling strategy was guided by the principle of maximum variation, enabling the identification of both shared patterns and context-specific experiences within the study population. The final sample comprised 16 women and 4 men. This composition reflects the recruitment process rather than the makeup of the older population; its implications for the analysis of gender are addressed under Data Analysis. Following the National Bureau of Statistics (2021) and the African Union Protocol on the Rights of Older Persons, persons aged 60 and above are defined here as older adults. This threshold is used for sampling only and is not taken to imply dependence.

### Data collection

Data were generated between April and May 2024 through semi-structured, in-depth interviews with a purposive sample of 20 older adults who had experienced the international migration of at least one adult child. Semi-structured interviews were selected for their ability to provide thematic consistency while allowing flexibility to accommodate participants’ individual narratives and experiences. The interview guide began with questions on participants’ sociodemographic characteristics, followed by open-ended questions exploring their children’s migration, including whether participants were consulted about the decision; how they understood and felt about their children’s departure; contact and support across distance, including communication, visits, and remittances; changes in caregiving arrangements and daily life; emotional well-being following migration; their sense of themselves as parents and their standing in the family and community; and sources of support beyond the immediate family, such as relatives, neighbors, and religious organizations. Probes were used to encourage elaboration, and each interview was closed by inviting participants to add anything not yet covered. Interviews were conducted in private settings conducive to candid disclosure, allowing participants to articulate their experiences in their own words and at their own pace. Interviews were conducted in English, Nigerian Pidgin English and/or Edo language. Interviews were audio-recorded with participants’ consent and transcribed verbatim. Interviews conducted in languages other than English were translated into English by language experts.

### Rigor, transparency, and credibility

Several measures were implemented to enhance the rigor, transparency, and credibility of this study. Credibility was strengthened through prolonged engagement and iterative questioning, which built rapport and ensured the authenticity of participants’ responses. Member checking was conducted by sharing emergent themes with a subset of participants for confirmation or clarification, ensuring that the findings reflected participants’ perspectives rather than the researcher’s impositions. Dependability was maintained through meticulous documentation of the research design, data collection procedures, and analytical decisions at every stage of the inquiry. A comprehensive audit trail—encompassing theme development rationale and reflective observations—ensured transparency and established that findings were systematically grounded in the data. Reflexivity was addressed through regular documentation of the researcher’s assumptions, positionalities, and potential biases. Recognizing the interpretative nature of qualitative inquiry, the researcher consciously examined how personal perspectives may have shaped analysis, bracketing preconceived notions to safeguard authentic representation of participants’ voices.

### Data analysis

The data were analyzed using IPA, a method well-suited to examine how individuals make sense of significant personal experiences. IPA’s dual emphasis on lived experience and the meanings participants attribute to those experiences made it particularly appropriate for exploring identity formation, emotional adaptation, and shifting social relationships in the context of family migration. The analytical process followed several systematic steps. Interviews were transcribed verbatim to preserve the integrity of participants’ expressions, and transcripts were read and re-read to establish deep familiarity with the data. Key phrases, emotional responses, and significant statements were identified and coded, with initial codes subsequently clustered into higher-order themes. This process facilitated the identification of patterns across four core areas: identity shifts, caregiving adaptations, emotional responses, and coping strategies.

Gender was approached as a relational and intersecting social position rather than as a binary attribute of participants (Mahler, 1999; Pessar and Mahler, 2003; Mahalingam et al., 2009). In keeping with the case-by-case analysis of IPA, we examined, within each account, how participants invoked gendered expectations of provision and authority over family decisions, and how these intersected with widowhood, income, health, and place of residence (Kaplan and Grewal, 1994; Purkayastha, 2010). Women’s and men’s accounts were analyzed within the same interpretive frame, and men’s accounts were not treated as a comparison or contrast group. Because mothers formed the majority of participants, the analysis speaks most fully to left-behind motherhood, which Mahalingam et al. (2009) show is shaped by intersecting positions rather than by gender alone. The fathers’ accounts are read for the gendered expectations they reveal, not as representative of older men’s experiences.

Given IPA’s inherently interpretative orientation, the researcher maintained ongoing reflexivity throughout the analysis, consciously examining personal influence on interpretive decisions to ensure transparency and analytical rigor. Pseudonyms were assigned to all participants to preserve confidentiality and anonymity.

### Ethical considerations

Ethical protocols were rigorously observed throughout the study to safeguard participants’ rights, dignity, and well-being. Prior to data collection, all participants provided informed consent via a consent form detailing the study’s purpose, data collection procedures, confidentiality protections, and their unconditional right to withdraw at any stage without consequence. Confidentiality was maintained through the anonymization of participant data and secure storage of all transcripts. Given the sensitive nature of discussions surrounding family separation, participants were encouraged to disclose only what they felt comfortable sharing. Interviews were conducted with empathy and cultural sensitivity, and breaks were offered when discussions became emotionally distressing. Throughout the process, the researcher remained attentive to participants’ emotional well-being, prioritizing their comfort alongside the integrity of data collection. Ethical approval for this study was granted by the Health Research Ethics Committee of the University of Benin Teaching Hospital, Benin City, Nigeria (ADM/E22/A/VOL.V11/148301225).

## Results

### Sociodemographic characteristics

The study sample comprised 20 older adults, 16 women, and 4 men, whose sociodemographic characteristics are presented in Table 1. More than half were aged 60 to 64 years, reflecting a relatively young cohort of older adults, and the rest were aged 65 to 69 or 70 and above. Most participants were currently married, and the remainder were widowed. Participants were drawn from rural, urban, and semi-urban communities, with rural residents forming the largest group. Educational attainment was generally low: most participants had completed only primary education, and a few had attained secondary or post-secondary education. Small-scale trading was the most common occupation, followed by housework and farming, reflecting the predominantly informal economic participation of older adults in this setting. Most participants reported no physical disability, although a few lived with paralysis or speech impairment. Similarly, most reported no chronic illness, while a few reported hypertension or rheumatoid arthritis. Every participant had at least one child abroad. For many, several children had migrated, and two reported that all their children lived abroad. Disaggregation by sex reveals that the structural profile of this left-behind population is itself gendered. Unremunerated domestic work was confined entirely to the female participants, with five women reporting their occupation as housewife and no male participant appearing in this category, indicating that the absence of independent earnings was an exclusively female condition within the sample. Farming, by contrast, accounted for half the male participants but only two of the 16 women. Educational disadvantage was likewise unevenly distributed: three of the four men had only primary education, compared with just over half of the women, and seven women had attained secondary or post-secondary education. Both participants reporting the migration of all their children were women, as were both participants living with rheumatoid arthritis. Marital status and residence, however, were distributed in broadly similar proportions across women and men, indicating that gendered differentiation within this sample operated principally through economic position and educational attainment rather than through household composition.

**Table 1:** Sociodemographic variables.

Variable	Categories	Female (*n* = 16)	Male (*n* = 4)	Total (*N* = 20)
Age	60–64	9	2	11
	65–69	4	1	5
	70+	3	1	4
Marital status	Married	12	2	14
	Widowed	4	2	6
Residence	Rural	7	2	9
	Semi-urban	3	1	4
	Urban	6	1	7
Highest level of education	Primary	9	3	12
	Secondary	4	0	4
	Post-secondary	3	1	4
Occupation	Farming	2	2	4
	Business	9	2	11
	Housewife	5	0	5
Disability status	None	14	3	17
	Speech impaired	1	0	1
	Paralysis	1	1	2
Chronic illness	None	12	3	15
	High blood pressure	2	1	3
	Rheumatoid arthritis	2	0	2
Number of children abroad	1-2	6	2	8
	3–5	5	1	6
	6-7	3	1	4
	All children abroad	2	0	2

### Migration as a social determinant reshaping care, well-being, and identity among left-behind older adults

The migration of children had wide-ranging consequences for the care, well-being, and identity of left-behind older adults. Some participants’ initial period following a child’s departure was frequently marked by emotional disorientation and difficulty adjusting to an altered domestic reality. As one participant recounted:

*…The experience at first wasn’t a sweet one, as it took me a long time to adjust to them not being around. When they were here, you know that your children are around you, and that all of you are together. But when they leave, things will not be the same again, as family will no longer be together in one place. But I have come to live with it because you cannot tell them to come back because they have gone there to look for a better life. But now, I am used to it, but it is not easy…*—Mrs. Mercy, Widowed, 70 Years Old

The framing of the experience as “not a sweet one” conveys not merely inconvenience but a deeper sense of emotional deprivation and disruption of the familiar social order. The prolonged adjustment period she describes suggests that the absence of children does not simply constitute a logistical loss but fundamentally unsettles the older adult’s sense of relational security and daily meaning. Also, for this widowed woman, a child’s migration removes not a supplementary source of companionship but the principal remaining one, meaning that the relational rupture described here compounds an earlier loss rather than occurring in isolation. Her observation that “you cannot tell them to come back because they have gone there to look for a better life” shows the separation being accepted as the price of her children’s prospects, and her account suggests that separation is an ongoing process of emotional adaptation rather than a single event.

Yet migration did not uniformly produce relational rupture. For some participants, physical distance was mediated by sustained emotional connection, facilitated through communication and transnational visits. A participant observed:

*…I am very close to two of my children abroad, I even visited last 2 months for my daughter’s graduation in the United Kingdom. So even though they are not here physically with me, we are still together because they are my children. I was very happy when I go visit (brings out her phone and shows the pictures) for my daughter’s graduation. Ahhh, I am very happy. Even though they are not here, I still feel them in my spirit never minding the distance…*—Mrs. Omorodion, Married, 68 Years Old

This account illuminates the capacity of transnational family bonds to transcend geographical separation. Traveling to witness her daughter’s graduation represents not merely a visit but a reaffirmation of parental presence and relational continuity across borders. Her remark that she still feels her children “in my spirit” shows closeness sustained through emotional and spiritual presence rather than co-residence. The milestone event of graduation further signifies that older adults remain active participants in their children’s lives despite physical distance, challenging narratives that frame migration exclusively as familial dissolution. Her ability to travel to the United Kingdom, however, points to material resources that not all participants share, suggesting that the compensatory potential of transnational contact is stratified.

This material dimension of migration also featured prominently in another participant’s account, particularly the role of financial remittances in enhancing access to healthcare and improving overall well-being. Accordingly,

*I know my condition is not an easy one, but I thank God. My daughter is trying her best. She buy(s) me medicine and make(s) sure I go to the hospital for check-up. At my age, we are now facing up, waiting for the day God will call us…Apart from being handicapped [paralysis], I am well taken care of, and my health has improved with good medical care through my daughter’s support. It is true I have this challenge, but at least, I am taken care of, and God is helping me…*—Mr. Oshioke, Widowed, 75 Years Old

The juxtaposition of physical disability with improved health outcomes in this account is analytically instructive. Despite his physical vulnerability, this participant’s narrative conveys a sense of security and dignified care—conditions made possible through his daughter’s remittances. This suggests that financial support from migrant children can function as a compensatory mechanism, partially offsetting the absence of direct caregiving. Also, his framing of being “well taken care of” despite his condition reflects a reconstruction of care that transcends physical proximity, repositioning the migrant child as a provider of care across distance rather than an absent figure. His references to God and to “waiting for the day God will call us” also place this care within a religious understanding of later life.

Beyond material well-being, migration reshaped some older adults’ social identities and community standing in significant ways. For some participants, having children abroad conferred elevated social status and community recognition, fundamentally redefining their social roles. This was illustrated as follows:

*…My sister, it is not only about what you have in your house…In this environment we live in, ever since the migration of my first daughter, people come to us for support, and we give them the little we can. We have never lacked support… Sometimes, people come to you because they need help, and in this life, if you have, even if it is small, give…We give foodstuff like garri, rice, tomatoes, even oil, salt pepper, name them…This is how we live here. Everybody cannot have everything but we try to help with what we have, and the people know.*—Mrs. Evukhowhiroro, Married, 65 Years Old

This narrative reveals how a child’s migration can catalyze a transformation in the older adult’s social identity. The capacity to extend support to neighbors and community members, enabled by her daughter’s remittances, repositions this participant from a potential recipient of care to an active provider within her social network. Her insistence that “this is how we live here” also situates this giving within local norms of reciprocity, so that remittances enter existing community obligations rather than simply conferring status. In this sense, the migrant child’s departure enhances rather than diminishes the parent’s social presence, illustrating the sometimes counterintuitive ways in which migration reshapes identity.

### Migration as a socially constructed experience among left-behind older adults

Left-behind older adults did not passively experience their children’s migration but actively engaged in meaning-making processes through which migration was socially constructed, rationalized, and interpreted. These constructions were shaped by economic realities, cultural norms, familial dynamics, and individual emotional responses, producing a complex and often contradictory landscape of meaning. Specifically, participants’ accounts show how they made sense of migration through the constraints on their say in the decision, the mixed feelings the departure produced, and their turn to support beyond the family.

For some participants, migration was primarily constructed as an economic necessity—a pragmatic, if painful, response to conditions of material deprivation. One participant articulated this as follows:

*As a mother and grandmother, it is not easy. It is not easy at all…It is not an easy decision at first, but due to the situation at hand, not being able to feed nine children, I had no option than to allow one of them to leave in search of greener pastures. Even, the one that traveled born pikin [child] and I am the one taking care of the child as him (her) mama travel. When you get nine children and things are not easy, you begin to think on what to do…I did not want to leave my child to travel but the situation was difficult. It was tough. I have to accept that my child will travel because going away will give us a better life…*—Mrs. Osuji, Widowed, 60 Years Old

The phrase “I had no option” encodes a sense of structural compulsion that constrains parental agency in the decision to migration. By framing migration as an inevitability imposed by economic circumstance rather than a freely chosen departure, this participant constructs a narrative of collective sacrifice in which the family unit—rather than the individual migrant - bears the moral weight of the decision. The idiomatic expression “greener pastures” further situates migration within a cultural discourse of hope and possibility, suggesting that even within resignation, aspirational meaning is preserved. Her account also shows that migration added to her caregiving: as a grandmother, she now cares for the child her migrant daughter left behind, so that the burden of migration includes renewed care for the next generation in later life.

Alongside economic rationalization, participants expressed a complex duality of pride and emotional loss. Mrs. Ochelli captured this tension:

*…It was a good experience because my daughter migrated for a good cause…but I still miss her… When you get to my age and look back, you are happy when you see your children are making progress in life, doing something good. You can be happy for them. But that one does not mean say you do not miss them. You see my child [refers to the interviewer], you can be happy that your child is gaining progress and also at the same, miss them well well. It is just that I no longer a small child, I would have been crying sometimes I miss them (laughs)*—Mrs. Ochelli, Married, 62 Years Old

The tension in this statement, between affirming migration as “a good cause” and admitting to longing, shows how left-behind parents negotiate the meaning of separation. Her pride in her daughter’s opportunity and her sense of loss sit side by side, neither canceling the other out. She locates this ambivalence in later life itself: looking back from old age, her children’s progress is a source of happiness even as their absence is felt. The meaning of migration for this participant is therefore layered, combining cultural acceptance of the departure with personal grief.

Cultural and familial authority further mediated the social construction of migration, as evidenced in accounts where parental consent was subordinated to spousal authority. One participant revealed:

*I was not happy before with the decision from the beginning because that was not what I wanted because of too many stories we hear about abroad life…I never supported the decision, but since she got the approval of her father, I had no choice but to agree. Her father already agreed, what else can I do? I cannot continue to oppose what her father already accepted. You know men, they do not like someone to challenge them even as I am his wife. So I agreed, even though agreeing does not mean that I was happy about it. Sometimes, in a family, one person can make decision even though everybody does not feel okay about it.*—Mrs. Dupe, Married, 60 Years Old

This account illuminates the gendered power dynamics embedded within family migration decisions. The participant’s initial resistance, ultimately overridden by her husband’s approval, reflects a patriarchal structure in which the mother’s objection is rendered insufficient as grounds for preventing migration. Her objection was grounded in “too many stories we hear about abroad life,” reflecting widely circulated knowledge of the risks of migration in this setting, and her remark that “men… do not like someone to challenge them” shows her reading the decision through the gendered authority of the household. Her use of “I had no choice” mirrors the compulsion expressed by Mrs. Osuji, yet here the constraint is social and gendered rather than economic. Read together, the two accounts suggest that some left-behind parents experience migration as something that happens to them rather than a process in which they are empowered participants.

Migration was also constructed through the lens of disconnection and exclusion, particularly where children departed without parental knowledge or consent. One participant recounted:

*…My son migrated without informing me, and it took me a long time to accept it. I was worried about his safety. That thing pain me well well, for so many months I cry. As a parent, you are supposed to know what your child wants to do especially big decision like this one. A good parent will like to know what is happening with the child. My son was traveling, he did not tell me. And the travel no be Benin to Agbor or even Asaba or may be Ontisha but a very long distance, abroad. I worried that time, whether my child will be fine…He call me when he reach there but he did not tell me he wants to travel out. It take me time to accept this but so far he is okay, no problem. My only concern and worry was whether he was fine and let nothing happen to him*—Mr. Ayomi, Married, 60 Years Old

Mrs. Dupe’s objection was voiced and then overridden; Mr. Ayomi was not consulted at all. Both accounts describe a loss of standing in a decision participants expected to share, though its form differed: deference to a husband’s authority in one case, and exclusion by the child in the other. Mr. Ayomi grounds his expectation in parental duty (“as a parent, you are supposed to know”), and his months of crying show that the exclusion was felt as a wound to that role. With one account of each, we note this difference without generalizing about how mothers and fathers experience exclusion from migration decisions.

Also, this absence of prior communication represents a rupture in the relational covenant between parent and child, a covenant in which the parent expects to occupy a consultative, if not decisive, role in major life decisions. His contrast between a journey to nearby towns such as Agbor or Asaba and one “abroad” marks international migration as qualitatively different in the distance and risk it entails. The prolonged period of acceptance described here suggests that what is being processed is not merely the fact of migration but the symbolic meaning of exclusion. The expressed worry about safety further reveals enduring parental orientation toward protection and responsibility, underscoring that physical distance does not dissolve the psychological dimensions of parenthood for left-behind older adults.

Finally, in the absence of proximate family support, social and religious institutions emerged as critical sources of meaning, belonging, and practical assistance. One participant stated:

*…It has been the church I fellowship with that has been assisting. That is where I have been receiving the support I need most of the time. They are really trying for people, honestly. When you find yourself ina situation where you need help, the good people around you become important. For me, the church has been there to assist me…The Church is like a mama that says, “Come my pikin [child], You no dey [you are not] alone”. You see, when family members no fit [cannot] provide for you, an an old woman, it is the church I run to. They are very important*—Mrs. Ehigiator, Widowed, 60 Years Old.

This reflects a restructuring of support networks following migration. It also reflects an ongoing, sustained relationship of support rather than episodic intervention, suggesting that the church has assumed a quasi-familial function in the participant’s life. Her description of the church as “like a mama” shows that she understands this support in kinship terms: the congregation does not simply assist her but takes on a parental role. Her reference to herself as “an old woman” whose family members cannot provide further suggests that, for this widowed participant, the church fills a gap left by both migration and widowhood.

### Migration as a gendered phenomenon reshaping roles, identities, and vulnerabilities among left-behind older adults

The challenges associated with children’s migration were neither uniform nor gender-neutral. Gender is examined here through the expectations participants invoked of mothers, fathers, sons, and daughters, rather than as a comparison between women and men. When participants themselves invoked the migrating child’s gender, this proved consequential, though it was not systematically elicited. These gendered dimensions intersected with health, cultural expectations, economic vulnerability, and emotional coping. For some women, particularly widows, physical and emotional vulnerability is combined. One participant disclosed:

*Physically, I’ve been in and out of several hospitals…it has really been lonely without my daughter. I rely on my provision business to survive…*—Mrs. Felicia, Widowed, 60 Years Old

This account weaves together physical deterioration, emotional isolation, and economic precarity into a single narrative of compounded vulnerability. Repeated hospital visits signal not merely ill-health but a body under sustained stress. The admission of loneliness further reveals the depth of relational dependency in this widowed participant’s life, having lost spousal support. Her reliance on a small-scale provisions business as a survival mechanism underscores the economic precarity of left-behind older women, who may lack access to formal income or pension support. Taken together, this account presents a layered disadvantage in which widowhood, illness, and limited income combine.

Cultural expectations surrounding age and filial proximity further shaped the gendered experience of migration, particularly for mothers whose daughters departed at what was perceived as a premature age. A participant said:

*…My daughter was at a tender age when she relocated…it was a painful and sad experience for me because I never gave my consent. When you think about your child so young and leaving home, you will be worried. It is not something to take lightly to just agree with it, but children of these days are so wise, not like when we were growing up. They just prepare you for marriage, and you don’t leave your father’s house until you marry and move to your husband’s house. For me, I did not agree, but she still go abroad…*—Mrs. Abiehode, Married, 60 Years Old

The descriptor “tender age” encodes a cultural norm in which daughters are expected to remain within the familial orbit until a socially recognized threshold of maturity is reached. Her contrast with her own youth, when daughters did not “leave your father’s house” until marriage, shows the distress being measured against a generational norm in which a daughter’s departure was tied to marriage rather than to migration. The participant’s withholding of consent, and her characterization of the experience as “painful and sad,” reveals the emotional weight of perceived cultural transgression, wherein the daughter’s early departure is experienced not only as personal loss but as a breach of expectations about gendered filial conduct. Whether the departure of sons carries a comparably gendered charge could not be assessed here, as the sex of migrating children was not systematically recorded.

Economic pressures and the gendered risks borne by women within household economies also emerged as indirect catalysts for migration, as one male participant, widowed, revealed:

*…My late wife, when she was alive…traveled to village markets mostly on a bike…that led to her death in an accident, which made my daughter’s decision to migrate. What happened to my late wife is something that is now part of our family story. She used to travel to village markets, and then the accident happened. When you have experienced this kind of loss, but God forbid, agreeing to your child traveling abroad will just be the only way, even though it is difficult.*—Mr. Friday, Widowed, 75 Years Old

This account illuminates the consequences that gendered economic responsibilities can produce. The wife’s death, a result of the physical risks she assumed in sustaining the household economy, functions in this narrative as a precipitating event that sets the migration process in motion. Her death is presented not only as a personal tragedy but as “part of our family story,” one that made agreeing to his daughter’s migration seem “the only way.” The daughter’s subsequent migration, framed here as a response to this loss, suggests that migration decisions are sometimes driven not by aspiration alone but by the disruption of existing family structures, particularly when that disruption is rooted in gendered economic vulnerability.

The migration of children also disrupted established gender roles for some older men, generating experiences of dependency that conflicted with culturally constructed masculine identities. One male participant reflected:

*…I was forced to depend on my daughter for support, something I never thought would happen as a man. As a man, you have some expectations of yourself, no matter how small. You want to be able to stand on your own and take care of your responsibilities. So when situation changes, I had to depend on my daughter. This is not something you imagined for yourself but I am grateful I have a good daughter, but this is still not what I expected my life to be…*—Mr. Iyengumwena, Married, 68 Years old

The phrase “as a man” is pivotal, functioning as a marker of the cultural expectations against which this participant measures his current condition. His reliance on a daughter for support, a reversal of the normative intergenerational care direction and a departure from masculine self-sufficiency, is experienced as a form of identity disruption. Yet he also expresses gratitude for “a good daughter,” so that dependency is experienced with both appreciation and unease. This account suggests that migration not only reorganizes practical care arrangements but can also unsettle gendered self-concepts, at least for this father, for whom independence and provision were central to his sense of self.

Gender also featured in how participants understood emotional coping, as one participant observed:

*…Women cope better and move on faster than the men. From what I have seen, women can find a way to continue their life even when things are difficult. May be because I am from Edo and we are hardworking, always have something to occupy our time like going to village market to buy things and selling them in the market. We women may feel it that our children are absent and we miss them, but we still have to hustle too. I think men find it difficult to adjust. I don’t know why (laughs) but may be because we their wives are not home 24/7 to sit with them. That is how I see it although another person may have their own experience…*—Mrs. Obazee, Married, 60 Years old

While this observation reflects a lay perception, its value lies in what it reveals about the social understanding of gendered resilience. Her own explanation points to women’s continued economic activity (“we still have to hustle”) and to Edo women’s market trading, which keeps them occupied; she also suggests that men may find adjustment harder when their wives are not at home. She presents this carefully as her own view, acknowledging that “another person may have their own experience.” Her account thus links coping less to gender as such than to the gendered organization of work and time.

Finally, the mental health consequences of migration-induced isolation were raised by participants. One participant observed:

*…Older people like us should never be left alone, there should be someone or something that can make us busy so our brain [mental health] should not be affected. When an older person is always alone without anything to do, it can affect how the person feels. We need people around us. We also need activities to be doing to keep us busy. It is not about giving an elderly person food, but we need companionship and something to occupy our mind, so we do not become lonely. You know they say idle mind is a devil’s workshop. At our age, our well-being and not being alone is important…*—Mrs. Aigbe, Married, 70.

This statement emphasizes keeping older adults “busy” and reflects an intuitive understanding of engagement as a protective factor against the psychological effects of prolonged isolation. Her insistence that “it is not about giving an elderly person food” indicates that she sees well-being as social as well as material, a distinction that remittances alone do not address.

Across the three themes, gendered expectations shaped how participants experienced and interpreted their children’s migration. These expectations appeared in women’s and men’s accounts alike: in Mrs. Dupe’s deference to the father’s approval, in Mrs. Abiehode’s concern that her daughter left at a “tender age,” in Mr. Friday’s account of the risks his wife bore in sustaining the household, and in Mr. Iyengumwena’s unease at depending on his daughter “as a man.” Gender operated in combination with widowhood, income, and health rather than independently of them.

## Discussion

The findings of this study are consistent with existing evidence on the consequences of international migration for left-behind older adults, and extend it to Edo State, Nigeria, contributing empirical depth to a body of literature that has historically privileged the experiences of migrants over those who remain. Read through the transnational social field perspective, they show older parents as participants in, rather than residues of, transnational family life (Levitt and Schiller, 2004).

The sociodemographic profile of participants helps situate the findings. Women comprised the majority of the sample, which reflects recruitment rather than population composition. Widowhood did not clearly differentiate participants. Access to independent income appeared more consequential: the five participants who reported unpaid domestic work were all women, and they depended wholly on remittances. Most participants relied on informal trading, subsistence farming, or unpaid domestic work. These livelihoods offer limited returns and may leave participants exposed if remittances are interrupted. Educational attainment was generally low, and the concentration of rural residents may compound these disadvantages, given the restricted access to healthcare, communication infrastructure, and formal social services that rural older adults in sub-Saharan Africa face (Ani and Batisai, 2024; Ani, 2014).

The emotional and psychological dimensions of the left-behind experience emerged as among the most significant findings of this study. Consistent with existing scholarship, the departure of children generated feelings of loneliness, emotional strain, and identity disruption among participants (Adhikari et al., 2011; Ebimgbo and Okoye, 2024; Abas et al., 2009). However, the findings also reveal the adaptive strategies of left-behind older adults, who actively engaged in meaning-making processes to reframe migration in ways that preserved psychological equilibrium. The rationalization of children’s departure as an economic necessity, a familial sacrifice, or a pathway to collective advancement reflects a strategy through which older adults reconcile the tension between personal loss and the social acceptance of migration as progress. This aligns with observations from comparable high-migration contexts, where left-behind individuals report more positive psychological outcomes when migration is collectively framed as a shared family investment in future well-being (Ebimgbo et al., 2022).

Participants’ accounts of migration as a necessity or as “a good cause” (Mrs. Osuji; Mrs. Ochelli) recall Gardner’s (2002) demonstration that older people make sense of migration through narratives of the life course. Gardner’s elders, however, were migrants themselves. Our participants narrate a migration they did not undertake but whose consequences they live with, which positions them as narrators of others’ mobility rather than their own. Their mixed feelings of pride and loss also correspond to what Luescher and Pillemer (1998) term intergenerational ambivalence, which reflects competing social expectations rather than individual indecision (Connidis and McMullin, 2002): in this setting, a child’s success abroad brings pride and status to the family (Akhigbe and Effevottu, 2023), while children are also expected to remain close to aging parents.

The role of community and religious institutions as surrogate support networks constitutes a particularly salient finding. In the absence of proximate family care, some participants drew on church communities and social networks to meet both practical and emotional needs. This is consistent with studies in south-east Nigeria showing that church-based organizations are an important source of support for older adults (Ebimgbo et al., 2018; Ukeachusim et al., 2023), although such support may be available only to members of particular congregations (Ebimgbo et al., 2018). It is also consistent with evidence that in contexts where formal social protection is absent or inadequate, informal community structures, particularly faith-based organizations, may function as an important institutional infrastructure sustaining older adults’ well-being (Kodzi et al., 2011; Ebimgbo et al., 2018; Ukeachusim et al., 2023). Mrs. Ehigiator’s description of her church as “like a mama” also illustrates that family support is dynamic and extends beyond the household and the parent–child relationship (Jarrett and Burton, 1999). In Edo’s extended family system, the church may take on functions that kin would otherwise perform, reflecting a reconfiguration of who counts as family in the absence of migrant children.

Participants’ continued closeness to children abroad, through visits, communication, and financial support (Mrs. Omorodion; Mr. Oshioke), echoes the well-documented capacity of transnational families to sustain care across distance (Hondagneu-Sotelo and Avila, 1997; Baldassar and Merla, 2014). Boehm (2012) similarly shows that gender and family relations are reworked across borders for those who remain as well as those who leave, and that the conditions of migration, including its legal precarity, shape family life on both sides. Mrs. Dupe’s fear of “stories…about abroad life” and Mr. Ayomi’s worry for his son’s safety suggest that such risks are felt by parents at home as well. What differs here is the direction of care. The transnational motherhood literature centers on migrant parents caring for children left in origin countries. In this study, it is aging parents who remain and adult children who provide care from abroad. Yet the two directions can meet: Mrs. Osuji now raises the child her migrant daughter left behind, occupying the grandmother’s position on which transnational motherhood often depends. Left-behind transnational parenthood thus extends existing accounts by showing parenthood maintained from the origin side, with support flowing upward across generations and care obligations sometimes flowing downward again.

Mrs. Evukhowhiroro’s account of becoming a source of support for neighbors is consistent with evidence that migration reshapes status in origin communities, both through money and through the social remittances that accompany it (Levitt, 1998; Ebimgbo et al., 2022; Akhigbe and Effevottu, 2023). Her account adds that this standing is expressed through local norms of reciprocity (“this is how we live here”), and our data suggest a gendered dimension to it: for older women with little independent occupational standing, remittances from children abroad may offer a basis for community recognition that would otherwise be largely unavailable.

The gendered norms that shaped participants’ experiences recall findings from immigrant communities. Dasgupta (1998) shows how expectations of daughters, in particular, carry the weight of cultural continuity among Asian Indian families in the United States. Mrs. Abiehode’s distress that her daughter left at a “tender age,” measured against a norm in which daughters remained in their father’s house until marriage, suggests that similar expectations of daughters operate in origin communities, where a daughter’s departure can be experienced as a breach of these norms. Mahler (1999) argued that migration transforms gender relations within origin households, and the accounts of Mrs. Dupe, overruled by the father’s approval, and Mr. Iyengumwena, unsettled by depending on his daughter “as a man,” show such relations being renegotiated in later life. In line with intersectional approaches (Mahalingam et al., 2009; Purkayastha, 2010), these experiences were shaped by gender in combination with widowhood, income, and health, not by gender alone; Mrs. Obazee’s explanation of women’s coping through market work points in the same direction.

Reimagining, however, was not available to everyone on equal terms. The forms of reconstruction that restored standing or closeness depended on remittances, the means to travel, and supportive institutions. Where these were absent, most visibly among widowed women managing illness alone, participants described the burden of migration without its compensations. Being left behind and being reimagined are therefore not alternative outcomes but coexisting ones. The balance between them was shaped by gender, marital status, health, and material resources. This is the central contribution the study seeks to convey through the concept of left-behind transnational parenthood: migration imposes a social burden on those who remain, and the capacity to reconstruct identity in response to that burden is itself unequally distributed.

The health implications of migration for left-behind older adults, while partially ameliorated by remittance-funded access to medical care, remain a significant concern. Participants managing chronic illness or disability in the absence of proximate family support face compounded challenges, as caregiving needs go unmet while medical expenditures increase. Comparative evidence from Bangladesh and Mexico similarly documents the intensification of healthcare dependency among left-behind older adults in high-migration contexts (Kuhn, 2005; Antman, 2010). Taken together, these findings suggest that the consequences of international migration extend beyond the migrant and the receiving context, reshaping the social, emotional, economic, and identity landscapes of those who remain.

Although this study was not designed to evaluate interventions, several implications follow from participants’ accounts. First, the women who reported unpaid domestic work depended wholly on remittances, so any interruption would remove their only source of income; social protection measures such as pensions and cash transfers could reduce this dependence. Second, participants managing illness or disability, such as Mrs. Felicia and Mr. Oshioke, relied on remittances to cover medical costs, underscoring the value of accessible, community-based healthcare for older adults whose children live abroad. Third, some participants’ reliance on church networks indicates that faith-based institutions already function as a care infrastructure for left-behind older adults (Ebimgbo et al., 2018; Ukeachusim et al., 2023). These institutions could be recognized as partners in local support efforts, bearing in mind that their support may reach only members of particular congregations (Ebimgbo et al., 2018). Fourth, Mrs. Aigbe’s call for companionship and activity, rather than food alone, underscores the value of community-based activities in countering isolation. Finally, because gendered expectations shaped participants’ experiences in different ways, including one father’s unease at acknowledging his dependence, support that assumes a single left-behind experience, or that relies on older adults seeking help voluntarily, may not reach all of them equally.

## Conclusion

This study examined how the international migration of children reshapes the care, well-being, and identity of left-behind older adults in Edo State, Nigeria. The findings point to a multidimensional process of adaptation, vulnerability, and identity reconstruction shaped by intersecting factors of gender, economic position, geographic location, and health status.

While financial remittances serve as critical lifelines, they can simultaneously generate dependencies that expose older adults to fragility, restructuring intergenerational caregiving and reversing conventional family dynamics. For at least one father, this reversal unsettled his sense of himself as a provider. Yet the findings equally resist a purely deficit-oriented framing: participants described ways of adapting, constructing meanings around migration that preserved purpose, pride, and social relevance, with religious and community institutions acting as surrogate support systems. For some of the older women in this study, remittances further afforded a basis of community recognition that their occupational positions did not otherwise provide. Being left behind and being reimagined were not alternative outcomes but coexisting ones, and the capacity to reconstruct identity in response to migration was itself unequally distributed. These findings illustrate left-behind transnational parenthood: parenthood, care, and standing reorganized from the origin side of the transnational field.

Ultimately, while migration generates economic opportunities for children abroad, it imposes under-acknowledged burdens on those who remain, burdens that are gendered, structural, and psychologically complex. This study contributes empirical evidence from a high-migration sub-Saharan African context, relevant to the African Union’s and the United Nations’ commitment to leaving no one behind.

## Study limitations

The most significant limitation of this study concerns the composition of the sample, which comprised 16 women and 4 men. Gender was analyzed through the expectations participants invoked rather than through comparison of women and men, and the four fathers’ accounts are illustrative rather than representative of older men’s experiences; the gendered observations reported here require exploration in samples of more balanced composition. Data on the sex of migrating children were likewise not systematically collected, precluding analysis of whether the departure of sons and daughters carries differing significance for those who remain, a question of evident relevance given cultural expectations differentiating filial obligation by sex.

Beyond this, the qualitative design and regional specificity of the sample limit the generalizability of the findings to Edo State’s particular cultural, economic, and social context. The purposive sample of 20 participants may not capture the diversity of experiences among left-behind older adults across broader sub-Saharan African contexts. Social desirability bias also poses a potential limitation, given the sensitive nature of discussions about family migration; however, deliberate efforts were made to foster a supportive, non-judgmental interview environment to encourage candid disclosure. Future research should employ larger, longitudinal, and mixed-methods designs across diverse sub-Saharan African settings, incorporating purposively gender-balanced sampling and systematic data collection on the sex of migrating children.

Similarly, the reasons for migration and the length of time children had lived abroad were neither used as selection criteria nor systematically recorded. Participants’ accounts indicate that children had migrated for employment, for study, or for a study that led to employment, and the left-behind experience may differ across these pathways and with the duration of separation. Nor was the legal status of children’s migration recorded; given the prominence of irregular migration from Edo State (Beber and Scacco, 2022), parents’ experiences may differ depending on whether children traveled via regular or irregular routes. Future studies would benefit from stratifying participants by migration type and specifying a minimum period abroad. Lastly, the study focused on the parent–child relationship. In Edo’s extended family system, siblings, grandchildren, and other kin may take on caregiving that migrant children cannot provide, and the roles of these relatives were not examined systematically.

## Data Availability

The raw data supporting the conclusions of this article will be made available by the authors, without undue reservation.
